# Determination of Optimal Diagnostic Cut-Offs for the Naval Medical Research Center Scrub Typhus IgM ELISA in Chiang Rai, Thailand

**DOI:** 10.4269/ajtmh.18-0675

**Published:** 2019-03-11

**Authors:** Meghna Phanichkrivalkosil, Ampai Tanganuchitcharnchai, Suthatip Jintaworn, Pacharee Kantipong, Achara Laongnualpanich, Wirongrong Chierakul, Daniel H. Paris, Allen L. Richards, Tri Wangrangsimakul, Nicholas P. J. Day, Stuart D. Blacksell

**Affiliations:** 1 Mahidol-Oxford Tropical Medicine Research Unit, Faculty of Tropical Medicine, Mahidol University, Bangkok, Thailand;; 2 Chiangrai Prachanukroh Hospital, Chiang Rai, Thailand;; 3 Department of Medicine, Swiss Tropical and Public Health Institute, Basel, Switzerland;; 4 Faculty of Medicine, University of Basel, Basel, Switzerland;; 5 Centre for Tropical Medicine and Global Health, Nuffield Department of Clinical Medicine, University of Oxford, Churchill Hospital, Oxford, United Kingdom;; 6 Viral and Rickettsial Diseases Department, Naval Medical Research Center, Silver Spring, Maryland;; 7 Department of Preventive Medicine and Biostatistics, Uniformed Services University of the Health Sciences, Bethesda, Maryland

## Abstract

In this diagnostic accuracy study, we evaluated data from 135 febrile patients from Chiang Rai, to determine the optimal optical density (OD) cutoffs for an in-house scrub typhus IgM ELISA. Receiver operating characteristic curves were generated using a panel of reference assays, including an IgM immunofluorescence assay (IFA), PCR, in vitro isolation, presence of an eschar, or a combination of these. Altogether, 33 patients (24.4%) were diagnosed as having scrub typhus. Correlation between positivity by IFA and increasing OD values peaked at a cutoff of 2.0, whereas there was little association between positivity by culture or eschar with increasing ELISA cutoffs—cutoffs of 3.0 and 4.0 were demonstrated to be optimal for the total absorbance of the OD at dilutions 1:100, 1:400, 1:1,600, and 1:6,400, for admission and convalescent samples, respectively. The optimal cutoff at a 1:100 dilution was found to be between 1.85 and 2.22 for admission samples and convalescent-phase samples, respectively. Sensitivities for the cutoffs varied from 57.1% to 90.0% depending on the reference test and sample timing, whereas specificities ranged from 85.2% to 99.0%. We therefore recommend a cutoff of around 2.0, depending on the sensitivity and specificity desired in clinical or epidemiological settings. The results demonstrate the ELISA to be a valuable diagnostic tool, suitable for use in resource-limited endemic regions, especially when used in combination with other diagnostic modalities such as the presence of an eschar.

## INTRODUCTION

Scrub typhus is a major cause of acute febrile illness in the Asia-Pacific region, accounting for up to 20–50% of febrile hospital admissions in endemic areas.^1–3^ *Orientia tsutsugamushi*, the causative agent in the region, is transmitted to humans through the bite of the larval stage of infected chigger mites.^4,5^ Clinical manifestations commonly observed include fever, eschar, rash, headache, myalgia, malaise, and regional lymphadenopathy. If left untreated, the disease may progress to multi-organ failure and death.^6^ With the exception of the eschar, scrub typhus is difficult to differentiate clinically from other febrile illnesses such as dengue, murine typhus, or leptospirosis. If diagnostic facilities are available, serological or molecular tests can be performed to enhance the clinical diagnosis, but both diagnostic methods have inherent weaknesses.^7^

The indirect immunofluorescence assay (IFA) historically has been considered the gold standard for laboratory diagnosis of scrub typhus.^7,8^ However, its application is limited in rural areas—where scrub typhus is especially prevalent—because of the level of technical expertise and infrastructure required.^7,9–11^ The ELISA is a more objective and convenient test to perform.^12,13^ Furthermore, it has been shown to be even more sensitive than the IFA.^13^ Other methods of laboratory diagnosis include PCR and in vitro isolation of *O. tsutsugamushi*—neither of which are feasible in resource-limited areas.^14^ Although a positive culture is confirmation of an infection, it is a laborious task that necessitates a biosafety level 3 facility.^14,15^ Moreover, it has been reported that the median time for detection of *O. tsutsugamushi* is 27 days.^16^ An indicative necrotic lesion known as an eschar is very useful at the time of disease presentation up until the lesion has completely healed for diagnosis (approximately 2 weeks), but its presence varies from 7% to 80% of cases.^6,17^

Herein, we investigate the use of a scrub typhus IgM ELISA for its utility in a highly endemic region, Chiang Rai in northern Thailand, to diagnose scrub typhus. To determine the assays applicability in Chiang Rai, we needed to ascertain the cutoff optical density (OD). Unfortunately, there is no consensus on a cutoff OD value for scrub typhus IgM ELISA that is applicable to Thailand. Previous diagnostic accuracy studies calculating cutoff OD values for ELISAs ranged from 0.2 to 1.3 at a 1:100 sample dilution, when compared with IFA or indirect immunoperoxidase assay as the reference standard.^14,15,18,19^ However, the conventional IFA IgM cutoff titer of ≥ 1:400 in the admission sample or a ≥ 4-fold rise to a minimum titer of ≥ 1:200 in the convalescent-phase sample has been suggested to have a high false-positivity rate.^20^ Using Bayesian latent class models, it was shown that antibody cutoff titers of ≥ 1:3,200 in the admission-phase sample or a ≥ 4-fold rise to ≥ 1:3,200 in the convalescent-phase sample provided the highest accuracy.^20^ In light of this, this study was conducted to determine the optimal OD cutoffs by performing receiver operating characteristic (ROC) curve analysis against a set of different reference diagnostic modalities for an in-house scrub typhus IgM ELISA.

## MATERIAL AND METHODS

### Samples.

Briefly, 135 hospitalized patients older than 15 years with acute undifferentiated fever of less than 2 weeks duration, no evidence of primary focus of infection, with three negative malaria blood smears, who provided written informed consent were consecutively recruited from August 2006 to October 2008 at Prachanukroh hospital, Chiang Rai, Thailand.^17,21^ Patient details including antibiotic therapy are presented elsewhere.^17,21^ Admission samples and, where possible, convalescent samples were collected prospectively. Ethical approval for this study was granted by the Ethics Committee of Chiang Rai Hospital, the Faculty of Tropical Medicine, Mahidol University, and the Thai Ministry of Public Health.

### Reference diagnosis.

The following reference diagnostic assays were performed to determine the final scrub typhus infection status of the patients included in the study: 1) in vitro isolation of *O. tsutsugamushi* from buffy coat samples performed using a previously described method^16^; PCR assays including the 56 kDa nested PCR assay,^22^ 2) 47 kDa–based quantitative real-time PCR (qPCR) assay^23^ and 3) GroEL-based qPCR assay^24^; and 4) the IFA based on *O. tsutsugamushi* pooled Karp, Kato, Gilliam whole cell antigens for scrub typhus.^19^ IgM antibodies were detected using IFA slides produced by the Australian Rickettsial Reference Laboratory (Geelong, Australia). Briefly, patient sera were serially 2-fold diluted from 1:100 to 1:25,600 and the endpoint was determined as the highest titer displaying specific fluorescence.^21^

Non-scrub typhus diagnosis was performed with dengue, Japanese encephalitis, murine typhus, and leptospirosis methodologies and criteria as presented elsewhere.^8,21^

### Scrub typhus IgM ELISA.

The antigens used for this study were whole-cell antigens of the Karp, Kato, and Gilliam strains of *O. tsutsugamushi* that were produced at the Viral and Rickettsial Diseases Department of the Naval Medical Research Center, Silver Spring, MD. Details of the *O. tsutsugamushi* ELISA antigen preparation including purification and formaldehyde inactivation are presented in Suwanabun et al.^19^ Antigen concentrations in the ELISA were optimized by block titration using homologous rabbit antisera using the method described by Dasch et al.^25^ Antigens were shipped and stored at a temperature between 2 and 8°C, with freezing avoided.

ELISA plates were prepared in the following manner: Two U-bottom 96-well microtiter plates coated with 1) *O. tsutsugamushi* strains pooled antigens consisting of 25 ng of Karp, 50 ng of Gilliam, and 100 ng of Kato strains and; 2) mock infected cell-lysate antigen in phosphate buffered saline (PBS) and incubated overnight at 4°C in a humid chamber. Serum samples were serially diluted from 1:100 to 102,400 in 1% skim milk/PBS buffer of which 100 μL from each dilution was transferred to each of the *O. tsutsugamushi* and mock infected cell-lysate ELISA plates and incubated at 37°C for 1 hour. Following incubation, the plates were washed four times with PBS/0.05% Tween 20 solution and bound IgM antibodies were detected by a 30-minute incubation at 37°C with anti-human IgM peroxidase conjugate (1:3,000 dilution, 100 μL per well; Invitrogen Corporation, Carlsbad, CA). Following washing a further four times, the two-component tetramethylbenzidine substrate (KPL, Inc., Gaithersburg, MD) was mixed in a 1:1 ratio and 100 μL was added to each well. The plates were then incubated in a dark chamber at room temperature for 30 minutes and 100 μL of 1 M hydrochloric acid was added to each well to stop the reaction. Plates were read at an optical wavelength of 450 nm (minus a reference OD value read at 650 nm) with a microtiter plate reader (Multiskan FC; Thermo Fisher, Singapore). The ODs from the mock antigen wells were subtracted as background absorbance to give a final average total absorbance (net OD or OD at 450 nm). Negative and positive control samples were used as a control of assay performance and were included in four wells each on each plate (two per control sample). Control sera were derived from pooled samples from northern Thailand with ELISA net OD < 0.2 (negative control; < 1:100 scrub typhus IgM IFA) and ELISA OD > 1.0 (positive control; ≥ 1:3,200 scrub typhus IgM IFA).

### Attribution of diagnosis.

Patients were classified as having scrub typhus on the basis of a modified scrub typhus infection diagnostic criteria^21^ which included one or more of the following: 1) Positivity in at least two of the three PCR assays targeting 56 kDa, 47 kDa, and groEL genes; 2) presence of an eschar; 3) successful in vitro isolation of *O. tsutsugamushi*. Furthermore, the following serological criteria were used: 4) an IFA IgM admission titer of ≥ 1:3,200 (*adm* ≥ 3,200) or 5) ≥ 4-fold rise to ≥ 1:3,200 in the convalescent sample (4-fold ≥ 3,200) that had previously been optimized as an IFA cutoff for Chiang Rai.^8^ Patients who did not have a convalescent sample collected were excluded from this study.

### Analysis.

To determine the most appropriate method to diagnose scrub typhus in Chiang Rai the following approaches were used.

#### Method 1: area under the receiver operator characteristic curve (AUROCC).

To determine the most appropriate ELISA OD cutoff, ROC curve analysis was performed using each component of the diagnostic criteria (i.e., PCR, IFA, etc.) as a reference comparator. To calculate a range of ELISA OD cutoff for admission samples alone or combined with convalescent samples, the *ROCTAB* command (Stata/IC 14.0 for Mac; Stata Corp., College Station, TX) and a range of diagnostic OD cut-offs were calculated with resultant sensitivity, specificity, and AUROCC values. The ELISA OD cutoff that provided the best balance between sensitivity and specificity was selected. Pearson correlation coefficients between individual reference assays and the scrub typhus IgM ELISA assay at various diagnostic cutoffs were also calculated.

#### Method 2: net total absorbance (NTA).

Using a method previously described,^26^ the net OD of the 1:100, 1:400, 1:1,600, and 1:6,400 serum dilutions were summed to obtain the NTA. Total absorbances of 1.000 or greater were considered evidence of scrub typhus and the titers were determined to be the inverse of the highest dilution that had an OD of 0.200 or greater. The cut-off of 1.000 was derived from a previous study that showed the benefit of using cutoff OD values of between 0.8 and 1.3 OD that gave the scientists the highest sensitivities and specificities of the *Orientia*-specific ELISA.^17^

## RESULTS

### Patient results.

Of 161 patients with acute undifferentiated fever, 135 (83.8%) had paired serology results as well as PCR, culture, and eschar data available, and were included in the study. The median days of illness before admission was 5 days, with an interquartile range (IQR) of 3–7 days. In the case of convalescent samples, where data were available (*n* = 82 patients), median days of illness before collection was 25 days (IQR: 22, 32) (Table 1). The median age of this cohort was 42 (IQR: 29–51), and 61% were male (Table 1).^17^

**Table 1:** Summary of available patient data

	Sample timing	Overall			ST positive		
		n*	Median	IQR	*n*	Median	IQR
Age	Admission	129	42	29, 51	31	43	32, 50
Gender	Admission	134	61% males		33	61% males	
Days of fever	Admission	130	5	3, 7	33	5	4, 7
	Convalescent	82	25	22, 32	33	27	21, 32

IQR = interquartile range; ST = scrub typhus.

* Full details unavailable.

**Figure 1.**
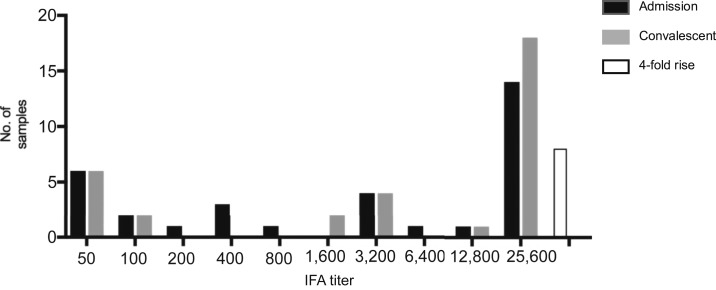
Immunofluorescence assay (IFA) titer distribution in admission and convalescent-phase samples of scrub typhus patients.

Using culture, eschar, PCR, or a combination of the three as reference comparators, 7 (5.2%), 14 (10.4%), 22 (16.3%), and 29 (21.5%) of the 135 patients were positive for scrub typhus, respectively (Table 2). Conversely, using the IFA diagnostic criteria, 25 (18.5%) cases were considered positive. Among these, 21 also had positive culture, eschar, or PCR results. Altogether, 33 (24.4%) patients were diagnosed as having scrub typhus according to the diagnostic criteria. For scrub typhus patients, the median days of illness before admission was 5 days (IQR: 4, 7) and convalescent was 27 days (IQR: 21, 32) (Table 1). The scrub typhus patients had higher IFA IgM titers in the convalescent-phase samples than acute samples (Figure 1), with a median titer of 1:25,600 (IQR: 1:850–1:25,600) and 1:3,200 (IQR: 1:150–1:25,600), respectively. Fourteen acute and 18 convalescent-phase samples demonstrated IFA IgM titers of ≥ 1:25,600, but as many as seven patients had titers ≤ 1:100 in both samples (six of these having ≤ 1:50) (list day of illness for acute and convalescent samples and prior antibiotic treatment to explain ab below limit of detection). The remaining patients had IgM titers of 1:200 (*n* = 1), 1:400 (*n* = 3), 1:800 (*n* = 1), 1:3,200 (*n* = 4), 1:6,400 (*n* = 1), and 1:12,800 (*n* = 1) in the acute sample, and IgM titers of 1:1,600 (*n* = 2), 1:3,200 (*n* = 4), and 1:12,800 (*n* = 1) in the convalescent-phase samples. Eight patients demonstrated a 4-fold rise to ≥ 1:3,200 between the acute and convalescent-phase samples.

**Table 2:** Overview of diagnostic indices of the optimal optical density (OD) cutoffs for IgM ELISAs, and patient positivity using the specified cutoff

Criteria		ELISA		Cutoff OD	Sensitivity	Specificity	Accuracy	AUROCC (95% CI)
		+	−					
Admission								
PCR	+	16	6	1.85	72.7	94.7	91.1	0.81 (0.67, 0.95)
	−	6	107					
Culture	+	4	3	2.16	57.1	88.3	83.0	0.63 (0.31, 0.94)
	−	15	113					
IFA adm ≥ 3,200	+	18	2	2.02	90.0	98.3	97.0	0.94 (0.84, 1.00)
	−	2	113					
IFA adm ≥ 3,200 or 4-fold rise ≥ 3,200 conv	+	20	5	1.85	80.0	98.2	94.8	0.89 (0.78, 1.00)
	−	2	108					
mSTIC	+	21	12	1.85	63.6	99.0	90.4	0.77 (0.65, 0.90)
	−	1	101					
Convalescent								
PCR	+	17	5	2.03	77.3	91.2	88.2	0.80 (0.66, 0.94)
	−	10	103					
Culture	+	5	2	2.22	71.4	85.2	84.4	0.67 (0.36, 0.98)
	−	19	109					
IFA adm ≥ 3,200	+	18	2	2.03	90.0	92.2	91.9	0.92 (0.82, 1.00)
	−	9	106					
IFA adm ≥ 3,200 or 4-fold rise ≥ 3,200 conv	+	22	3	2.03	88.0	95.5	94.1	0.90 (0.80, 1.00)
	−	5	105					
mSTIC	+	23	10	2.03	69.7	96.1	89.6	0.77 (0.64, 0.90)
	−	4	98					

AUROCC = area under the receiver operator characteristic curve; IFA = immunofluorescence assay; mSTIC = modified scrub typhus infection diagnostic criteria.

### The optimal ELISA diagnostic cutoff.

#### Method 1: AUROCC.

**Figure 2.**
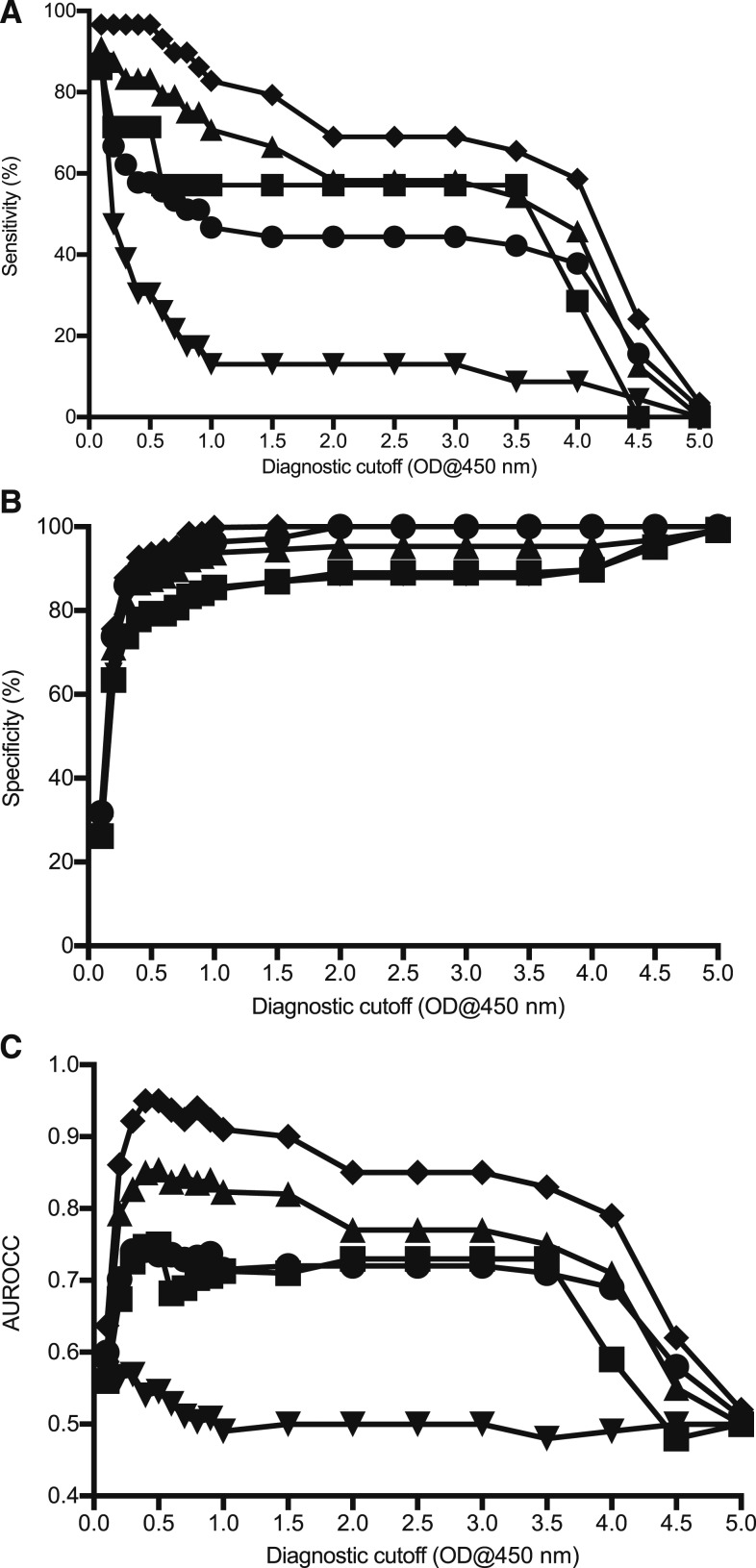
Overview of admission sample over the range ELISA cutoff optical densities (ODs) plotted for various diagnostic modalities of (▲) PCR; (▪) Culture; (▼) immunofluorescence assay (IFA) *adm* ≥ 3,200; (♦) IFA *adm* ≥ 3,200 or 4-fold ≥ 3,200; (●) modified scrub typhus infection diagnostic criteria. Sensitivity (**A**) and specificity (**B**) of the ELISA cutoffs, and correlation (**C**) between reference test positivity and ELISA positivity are shown.

In general, the cutoffs calculated using ROC for the admission samples had lower sensitivities as compared with the convalescent-phase samples but higher specificities (Figure 2A and B). Diagnosis based on *adm* ≥ 3,200 gave the best balance between sensitivity (90.0%) and specificity (98.3%), at a 2.03 admission cutoff. The optimal ODs of on-admission samples were all comparable between the different diagnostic modalities, being in the range of 1.85–2.16. The cutoffs were also similar for the convalescent-phase samples, ranging from 2.02 to 2.22. The optimal was 2.03, which gave a sensitivity and specificity of 88.0% and 95.5%, respectively, based on the admission IFA IgM titer of *adm* ≥ 3,200 or a 4-fold ≥ 3,200 diagnostic criteria. Using nonserological methods (bacterial detection methods or presence of eschar) as a reference comparator gave low sensitivity values, with the optimal ranging from 57.1% to 77.3% depending on the test and sample timing (Table 2), whereas using the combined criteria to diagnose patients gave cutoffs with relatively high specificities but low sensitivity values.

Correlations between reference assay positivity and ELISA positivity peaked at a cutoff of around 2.0 for all tests (Figure 2C), in agreement with the optimal cutoffs obtained using ROC curves (Table 2). Positivity using culture and eschar as the reference criteria had low (< 0.50) correlation with ELISA positivity (0.28–0.29 and 0.43–0.47 at a 2.0 OD, respectively), and this is reflected in the sensitivity and specificity results. Conversely, PCR and IFA results correlated well with ELISA results with IFA demonstrating the strongest relationship as would be expected for assays detecting the same analyte. There was a correlation of 0.88 between an ELISA admission sample cutoff of 2.0, and an IFA *adm* ≥ 3,200. This correlation was lower in the convalescent-phase samples (0.71 at a cutoff of 2.0).

#### Method 2: NTA.

**Figure 3.**
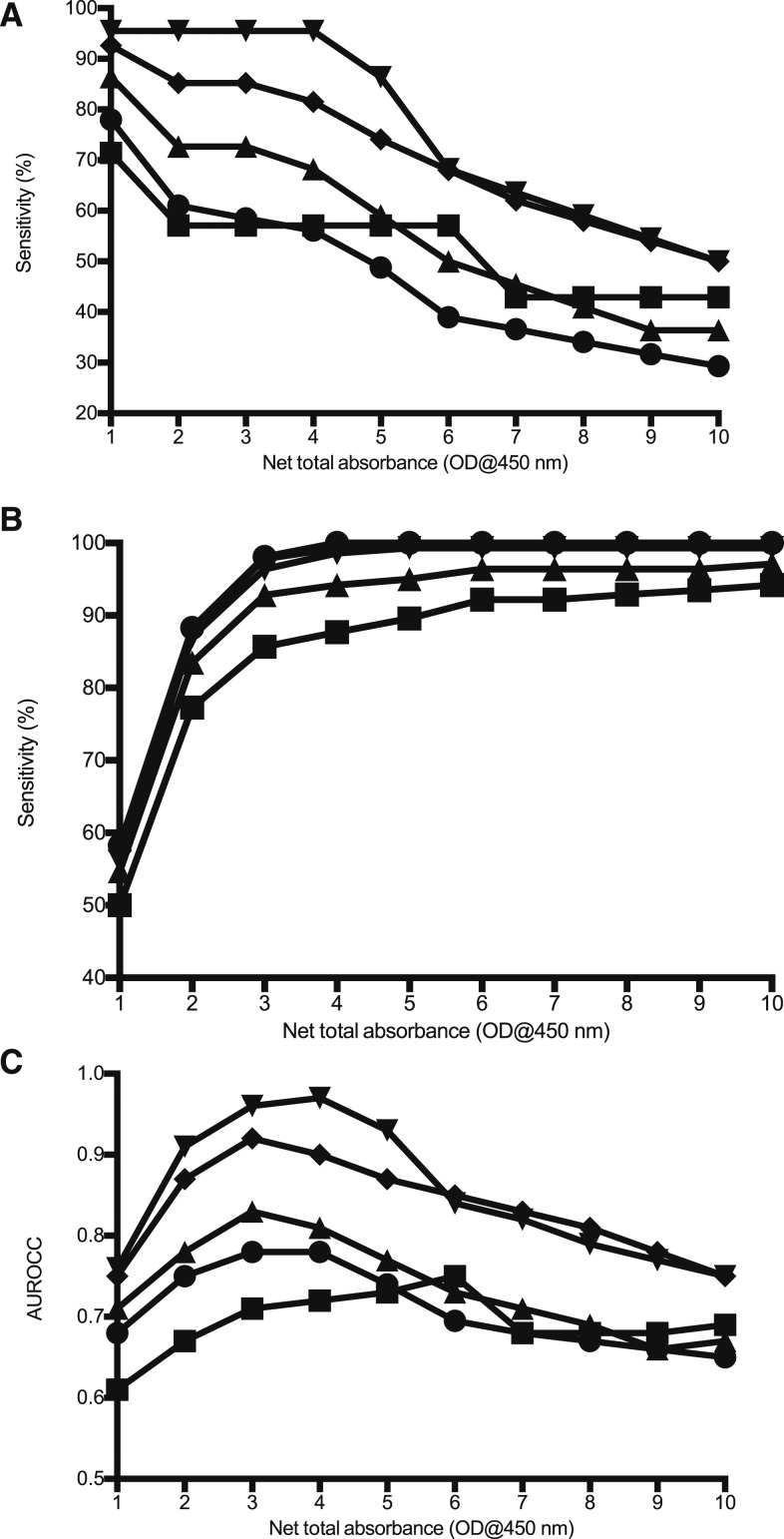
Overview of admission (samples over the range of net total absorbance cutoff optical densities (ODs) plotted for the various diagnostic modalities of (▲) PCR; (▪) Culture; (▼) immunofluorescence assay (IFA) *adm* ≥ 3,200; (♦) IFA *adm* ≥ 3,200 or 4-fold ≥ 3,200; (●) modified scrub typhus infection diagnostic criteria. Sensitivity (**A**) and specificity (**B**) of the ELISA cutoffs, and correlation (**C**) between reference test positivity and ELISA positivity are shown.

Net total absorbance analysis using the sum of the OD values demonstrated a cutoff of 3.0 (66.7% sensitivity, 99.0% specificity) and 4.0 (72.7% sensitivity, 96.1% specificity) to be optimal for the admission and convalescent samples, respectively (Table 3, Figure 3).

**Table 3:** Net total absorbance (NTA) analysis using the sum of the optical density (OD) values with corresponding sensitivity and specificity values

Sample timing	NTA OD cutoff	Sensitivity (%)	Specificity (%)	AUROCC
Admission	1.0	81.8	54.9	0.68
	2.0	66.7	90.2	0.78
	3.0	66.7	99.0	0.83
	4.0	63.6	100	0.82
Convalescent	1.0	81.8	41.2	0.62
	2.0	72.7	73.5	0.73
	3.0	72.7	90.2	0.82
	4.0	72.7	96.1	0.84

AUROCC = area under the receiver operator characteristic curve.

## DISCUSSION

In this study, we have determined an optimal IgM ELISA cutoff value for diagnosis of scrub typhus infections in Chiang Rai by performing ROC curve analysis on results utilizing admission and convalescent-phase samples.

The conventional IFA IgM cutoff titer of ≥ 1:400 in the admission sample or a 4-fold rise to ≥ 1:200 in the convalescent-phase sample may lead to a high rate of false positivity.^8,20^ Using the same reference samples from Chiang Rai as this present study, Bayesian latent class modeling was used to show that an admission titer of *adm* ≥ 3,200 or a 4-fold ≥ 3,200 in the convalescent-phase sample provided the highest sensitivity and specificity.^18^ A strong correlation was seen between increasing OD values and IFA IgM positivity using this criterion (0.79–0.82), thereby validating its use as a reference standard. Cutoffs of 1.854–2.222 were found to be optimal for the admission sample and convalescent-phase samples, in-line with the Blacksell et al.^12^ study, which found a cutoff of 1.474 to be optimal at a 1:400 sample dilution. Performance of the ELISA shows sensitivity ranging from 57.1% to 90.0% and specificities of 85.2% to 97.0%, depending on the reference criteria and sample timing. When using the total absorbance for serum dilutions 1:100, 1:400, 1:1,600, and 1:6,400 as cutoffs, previous studies have used a value of 1.0, for samples that pass a preliminary screen (OD ≥ 0.5 at a 1:100 dilution). These studies have been conducted in endemic regions such as Korea and India, as well as those with lower prevalence of scrub typhus, such as Peru.^26,27–29^ However, we show here that in endemic regions such as Chiang Rai, cutoffs of around 3.0 and 4.0 are more appropriate for admission and convalescent samples, respectively. The sensitivity and specificity values obtained using this method are comparable with those obtained using the single dilution cutoff titers. Despite a limited sample size, the results are likely to be representative of the Chiang Rai population, given the spread of age (median: 42, IQR: 29–51), and the proportion of males and females (61% males).

The cutoff values obtained were significantly higher than those normally used in studies conducted in Thailand and other endemic regions. This reflects the difference in reference cutoffs, which are conventionally set at an IFA IgM titer of ≥ 1:400.^14,15,19,20,30^ Oftentimes arbitrary OD cutoffs are applied, particularly in studies using InBios ELISA kits, as these provide control serum samples irrespective of the geographical location in which the study is being carried out. In such cases, the cutoff is often set at 0.5, for example in endemic regions of India, where the use of InBios kits is widespread.^31–34^ This is especially problematic when considering that high background antibody levels in such regions can lead to false positive results. In this study, the majority of IFA-positive samples (84%) were also positive by culture, eschar, or PCR, indicating the adm 3,200 plus 4-fold rise criteria to be specific and suggestive of a present infection. This is significant because IgM levels have been shown to remain elevated for 12 months after infection in most patients (K. Phakhounthong, in preparation). Four of the patients in this study were positive by IFA but negative by culture, eschar, and PCR, but this is expected, given the lower limit of detection of such methods—PCR positivity has been shown to be affected by treatment, and eschars can be present in as little as 7% of scrub typhus patients (42.4% in this study).^6,35^ In general, there is lower correlation between serology and non-serological diagnostic methods, and the latter are known to give false-negative results.^14,36^ This accounts for the lower sensitivity of the cutoffs calculated based on these tests (especially when combined) and illustrates the need to carefully select appropriate reference standards.

In scrub typhus patients, antibodies appear following the bacteremic phase. Hence, the disease may not be readily detectable by serology if the admission sample is taken during the earlier stages of infection (< 5 days), whereas it may be if using culture or PCR.^1,21^ Correspondingly, there were more patients positive by ELISA in the convalescent-phase samples, despite the use of higher cutoff values. This includes samples that were negative by the IFA reference criteria, suggesting that the ELISA may be superior for detection of scrub typhus. This could speak to the subjective nature of the IFA results, which requires skilled technicians to interpret. Interestingly, our results also revealed that higher OD values correlated with PCR positivity (0.62–0.66), suggesting that the stages of the disease may not be as distinct as previously thought.

Where possible, using a combination of assays—including bacterial detection methods—to diagnose scrub typhus is likely to provide the highest accuracy, given that in some cases serology does not indicate an infection. We found that in six patients diagnosed as having scrub typhus based on quantitative and conventional PCR, culture, or eschar positivity, antibody titers remained ≤ 1:50 in both acute and convalescent samples, highlighting the value of a combination-based approach in diagnosis. For example, another study demonstrated that using both immunochromatographic tests and a loop-mediated isothermal amplification PCR assay for diagnosis of scrub typhus increased sensitivity up to 20%, without a significant loss in specificity.^2^ Although PCR and in vitro isolation methods may not be feasible in resource-limited settings, the presence of an eschar may be a valuable indicator of scrub typhus infection, particularly when combined with serology. Moreover, it is likely to be fairly specific as other common causes of eschars such as anthrax and spotted fever group rickettsioses are not prevalent in Thailand.^36,37^

One limitation of this study is that the convalescent sample collection dates were not available. A confirmatory, retrospective diagnosis is often made based on a 4-fold rise between the acute and convalescent-phase samples.^7^ The main drawback to using this criterion is that adequate time is needed between collection of the two samples to demonstrate a rise in titer, and in some cases, as mentioned previously, titers may not rise at all.^7,38^ Delaying treatment for this long could result in a number of complications including systemic infection and multiple organ dysfunction; hence, diagnosis is based on acute samples.^6^ Furthermore, depending on the onset of illness, admission samples may already exhibit high antibody titers, in which case, a 4-fold rise may not be seen. In this cohort, only eight patients of the 33 scrub typhus–positive patients showed a 4-fold rise or more. Therefore, in clinical settings—where treatment should be administered as soon as possible—it is necessary to establish a single cutoff titer based on regional endemicity.

The results demonstrate ELISA to be a suitable alternative to the IFA, offering advantages over it such as reader objectivity and reproducibility.^1^ Correlation between the two exceeded 0.7, at the optimum cutoff of 2.0. In this study, pooled antigens of the Karp, Kato, and Gilliam strain were used. Fittingly, studies have identified predominantly Karp-type strains—and to a lesser extent Gilliam-type strains—around Thailand, although the strains in Chiang Rai have not been fully characterized.^39,40^ Furthermore, culturing native antigens requires a biosafety level 3 facility, making it less practical to perform in rural areas.^16,30^ ELISAs that use a recombinant 56-kDa antigen have been shown to be a highly sensitive and specific alternative.^15,30^

Here, we have shown the ELISA to be a valuable diagnostic tool, especially at a recommended cutoff of around 2.0, and a total absorbance cutoff of 4.0. These values can be adjusted to find an appropriate balance between sensitivity and specificity, depending on whether the test is being conducted from an epidemiological or diagnostic perspective. Considering the unusually high value of these cutoffs, there is a need to 1) validate the use of the cutoffs in Chiang Rai, and 2) re-evaluate the cutoffs used in other endemic regions to ensure they are considering background antibody levels.
